# Does low income effects 5-year mortality of hepatocellular carcinoma patients?

**DOI:** 10.1186/s12939-021-01498-z

**Published:** 2021-09-01

**Authors:** Dong Jun Kim, Ji Won Yoo, Jong Wha Chang, Takashi Yamashita, Eun-Cheol Park, Kyu-Tae Han, Seung Ju Kim, Sun Jung Kim

**Affiliations:** 1 Department of Health Administration and Management, Soonchunhyang University Graduate School, Asan, Republic of Korea; 2 Center for Healthcare Management Science, Soonchunhyang University, Asan, Republic of Korea; 3 Department of Internal Medicine, University of Nevada Las Vegas School of Medicine, Las Vegas, Nevada USA; 4 Department of Health Administration, College of Business, Texas Womenʼs University, Denton, TX USA; 5 Department of Sociology, Anthropology, and Health Administration and Policy, University of Maryland, Baltimore, MD USA; 6 Department of Preventive Medicine, Yonsei University College of Medicine, Seoul, Republic of Korea; 7 Institute of Health Services Research, Yonsei University College of Medicine, Seoul, Republic of Korea; 8 Division of Cancer Management Policy, National Cancer Center, Goyang, Republic of Korea; 9 College of Nursing, Catholic University of Korea, Seoul, Republic of Korea; 10 Department of Health Administration and Management, College of Medical Science, Soonchunhyang University, 22 Soonchunhyang-ro, Asan, 31538 Republic of Korea; 11 Department of Software Convergence, Soonchunhyang University, Asan, Republic of Korea

**Keywords:** Hepatocellular carcinoma, Liver Cancer, Low income, Mortality, Multi-level analysis, Cox proportional hazard model

## Abstract

**Background:**

In Korea, the universal health system offers coverage to all members of society. Despite this, it is unclear whether risk of death from hepatocellular carcinoma (HCC) varies depending on income. We evaluated the impact of low income on HCC mortality.

**Methods:**

The Korean National Health Insurance sampling cohort was used to identify new HCC cases (*n* = 7325) diagnosed between 2004 and 2008, and the Korean Community Health Survey data were used to investigate community-level effects. The main outcome was 5-year all-cause mortality risk, and Cox proportional hazard models were applied to investigate the individual- and community-level factors associated with the survival probability of HCC patients.

**Results:**

From 2004 to 2008, there were 4658 new HCC cases among males and 2667 new cases among females. The 5-year survival proportion of males was 68%, and the incidence per person-year was 0.768; the female survival proportion was 78%, and the incidence per person-year was 0.819. Lower income was associated with higher hazard ratio (HR), and HCC patients with hepatitis B (HBV), alcoholic liver cirrhosis, and other types of liver cirrhosis had higher HRs than those without these conditions. Subgroup analyses showed that middle-aged men were most vulnerable to the effects of low income on 5-year mortality, and community-level characteristics were associated with survival of HCC patients.

**Conclusion:**

Having a low income significantly affected the overall 5-year mortality of Korean adults who were newly diagnosed with HCC from 2004 to 2008. Middle-aged men were the most vulnerable. We believe our findings will be useful to healthcare policymakers in Korea as well as to healthcare leaders in countries with NHI programs who need to make important decisions about allocation of limited healthcare resources according to a consensually accepted and rational framework.

## Background

Mortality rates due to hepatocellular carcinoma (HCC) have tended to increase in many countries in recent decades [1]. In 2018, HCC was the second most common cause of cancer-related mortality in Korea, with a rate of 20.7 per 100,000 [2], which is two- to five-fold higher than in most European countries and the United States [1]. The Korean government has implemented many cancer management policies to identify and resolve these HCC problems [3]. However, the results of the policy were not equal among all HCC patients in Korea [4].

In Korea, HCC is the most common cause of cancer mortality in men aged 40–59 [3]. The consequent economic loss in this age group was estimated to be US$2.8 billion in 2014, which is the largest economic deficit associated with any type of cancer in Korea [3]. Individual-level biological and contextual factors, such as economic conditions, can affect HCC mortality rates [5, 6]. Low income, which is usually defined as the bottom 20% of the income distribution in a country [7], can increase the risk for mortality in HCC patients [5, 6]. Low income is linked to barriers to both formal and informal access to overall healthcare and, in turn, to HCC treatment [8]. Although there have been no nationwide studies on the effects of income on HCC mortality under the universal health system, a study of Ontario [9] residents showed that HCC patients in the lowest income quintile had a 10% higher HCC-related mortality rate than other groups [9].

In terms of socioeconomic factors, access to health insurance is a key factor that enables patients to benefit from the most current treatments [10]. In Korea, under the universal health system, health insurance coverage applies to all members of society. However, individual income is another factor that enables access to health care because, in 2015, the mandatory public health insurance covered only 64% of all healthcare expenditures, leaving 36% of these expenditures to be paid by private supplementary insurance or individuals [7]. Uncovered services included surcharges for specialists at general hospitals, new and high-cost diagnostic or therapeutic services, and private wards [11]. Standard coverage by the National Health Insurance (NHI) can be insufficient for Korean households in general and low-income adults in particular [12]. To increase the financial protection available in the event of catastrophic illness, the Korean government expanded the NHI coverage for cancer patients in 2005; however, the gap between the benefits available to low- and high-income cancer patients remains unchanged [11, 12].

Furthermore, although a few studies have examined the occurrence of and survival following diagnoses of breast, prostate, and colorectal cancer as a function of regional socioeconomic status (SES) [13–16], there has been no comparable HCC-related research. This study investigated the association between low income and HCC mortality at the national level in Korea in the context of community characteristics. We also evaluated whether this association differed by age and sex and estimated the HCC-related mortality risks associated with individual-level demographic characteristics.

## Methods

### Study population

**Fig. 1:**
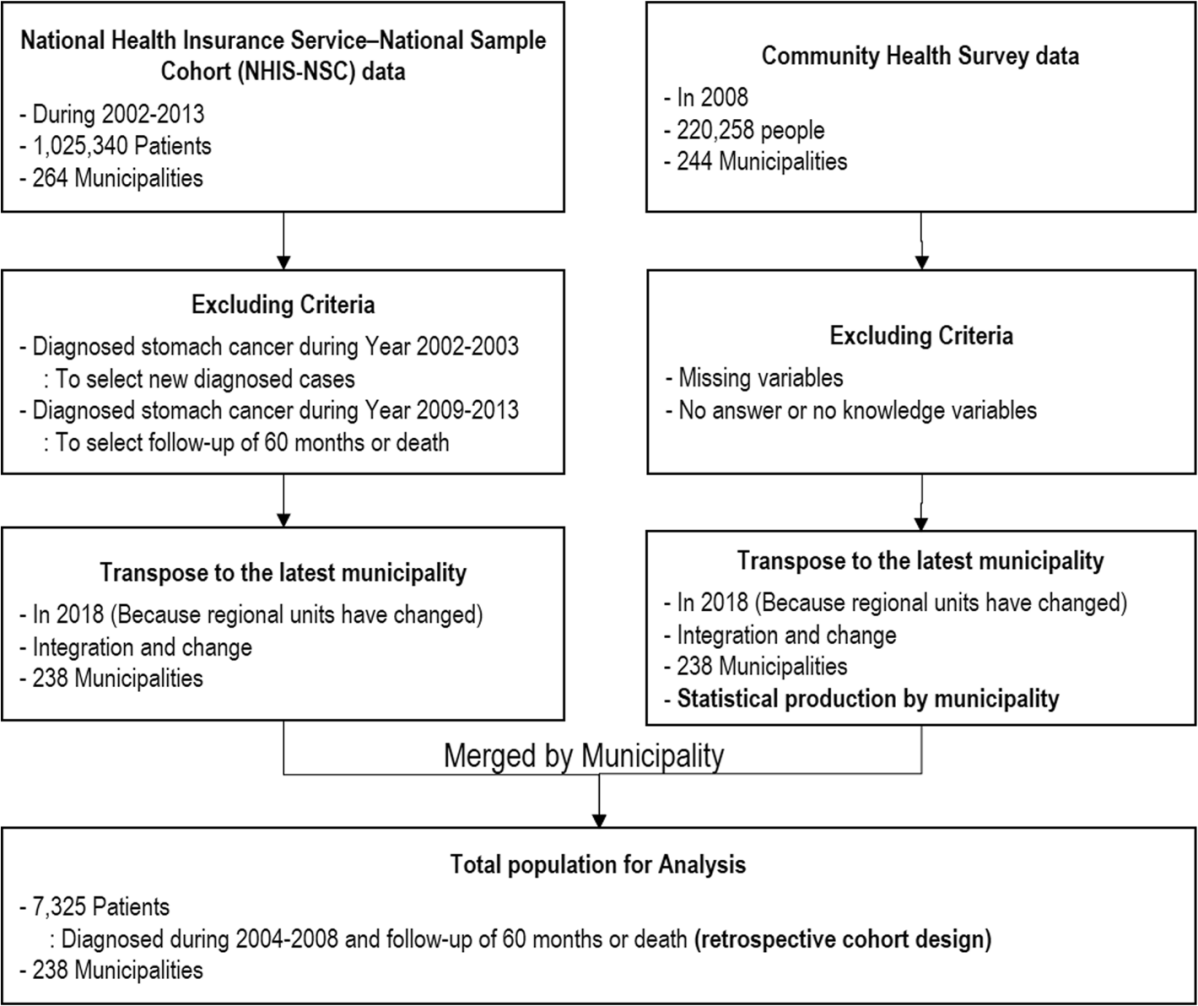
Flowchart of sample selection process

We used data from the Korean National Health Insurance Service-National Sampling Cohort (NHIS-NSC), which was collected based on a systematic sampling design in 2002–2013, to produce a nationally representative random sample of 1,025,340 individuals, as well as the 2008 Korea Community Health Survey (KCHS) involving data on 200,000 individuals obtained from 253 community health centers [17, 18]. To investigate the associations between individual- and community-level characteristics and survival of HCC patients, we first identified individuals diagnosed with HCC between 2002 and 2013 according to the International Classification of Diseases, version 10 (ICD-10): C22. Then we excluded patients diagnosed during 2002–2003 to ensure that our sample was restricted to newly diagnosed HCC cases under the assumption that, if an individual had no HCC diagnosis in the entire two-year period, then the first diagnosis of HCC from 2004 onward was new. This is because the first diagnosis should be distinguished, taking into account the long disease cycle of cancer. We also excluded patients diagnosed with HCC during 2009–2013 to further restrict the sample to only patients who were followed for 60 months because it is impossible to track the censoring that occurs during this period; this criterion covered patients who were diagnosed during 2004–2008. Then we transposed the dataset into a retrospective cohort design in which the unit of analysis was information from each HCC patient. These claims data consist of a single case of patient medical use. We summarized each case into one patient episode. After that, survival time was measured from the first diagnosis to the time of death, and patients who did not die were defined as survival. To evaluate each HCC patient’s community-level characteristics, we summarized each municipality’s characteristics from individuals of the KCHS, conducted by the Korean Center for Disease Control and Prevention [17, 18]. Furthermore, we matched individual- and community-level data and obtained data on the characteristics of 7325 new HCC patients and their respective 238 municipalities (Fig. 1). This study was reviewed and approved by the Institutional Review Board of Soonchunhyang University (2017–05-BM-014).

### Variables

The main outcomes were number of deaths and survival time from HCC over the course of a 5-year follow-up period. The index date was defined as the date of diagnosis. All-cause death data were included in the dataset; however, cause of death could not be identified. Individual-level variables were sex, year of HCC diagnosis, HCC etiology, patient age, income, and disability at diagnosis of HCC. Patient ages were categorized into the following groups (in years): 49 or younger, 50–59, 60–69, and 70 or older. We categorized income into NHI contribution quintiles: 1st quintile (20th or lower percentiles), 2nd quintile (21st–40th percentiles), 3rd quintile (41st–60th percentiles), 4th quintile (61st–80th percentiles), and 5th quintile (80th or higher percentiles) [17, 19]. Data on the status (physical disability or all-cause disability) and severity (grade 1 or 2, severe; grade 3–6, mild) of the disability were provided by the NHIS-NSC based on evaluations performed by the treating physician according to the specific guidelines established by the Korean government [17, 18]. The etiologies of HCC were defined as follows based on ICD-10 coding: HBV, hepatitis c (HCV), alcoholic liver cirrhosis, and other (non-viral non-alcoholic) liver cirrhosis. The KCHS analyzed the following community-level variables in 2008: current smoking rate, high-risk drinking rate, percentage of residents who walk for exercise, obesity rate, and percentage of college graduates. The current smoking rate was defined as the percentage of people who had smoked more than five packs (100 cigarettes) in their lifetime and who were currently smoking (smoked “daily” or “sometimes”). High-risk drinking was defined as consuming seven or more (men) or five or more (women) alcoholic drinks on the same occasion on at least 2 days within the past 7 days. The percentage of residents who walked for exercise was the percentage of people who had walked more than 30 min per day during the last week (7 days). The obesity rate was defined as percentage of people with a body mass index (BMI; kg/m^2^) greater than 25.

### Statistical analyses

We first examined the number and characteristics of individuals newly diagnosed with HCC during the 5-year study period. The frequencies and relative percentages were calculated for categorical variables, and χ2 tests were performed to examine differences in each variable by survival. The incidence per person-years and follow-up period were examined as a function of patient characteristics. Also, according to income level, the distribution of deaths and the person-years at deaths were calculated for each variable, and a χ2 test was conducted on the distribution. To investigate associations between patient characteristics and death from HCC, after testing the proportionality assumptions by the Grambsch and Therneau non-proportionality test and log (− log {S (t)}) plot, we used Cox proportional hazard models to estimate hazard ratios (HRs) and corresponding 95% confidence intervals (CIs). To identify the individual and community factors associated with the survival probability of HCC patients, we employed a multi-level survival model to include regional-level random effects in an individual model. In addition, subgroup analyses were performed by sex and age groups, trend analysis was performed according to decrease in income level by model, and the threshold for statistical significance was set at *p* < 0.05 (two-tailed) for all analyses. All statistical analyses were performed using SAS statistical software, version 9.4 (SAS Institute Inc., Cary, NC, USA).

## Results

### Patients and characteristics

Table 1 presents patient characteristics, number of deaths, and mean survival times. The number of new HCC cases from 2004 to 2008 was stable, with 4658 cases among males and 2667 cases among females. The 5-year survival proportion of males was 68%, and the incidence per person-year was 0.768; the female survival proportion was 78%, and the incidence per person-year was 0.819. The mortality proportion of the highest income group was 25%, while those of the 4th, 3rd, 2nd, and 1st quintiles were 26, 28, 31, and 35%, respectively (*p* < 0.001). Patients with liver cirrhosis had higher mortality rates and shorter survival times than patients without cirrhosis, but an inverse association was found for patients with HBV or HCV. Table 2 shows death information by income level. Table 2 presents, according to income level, the distribution of deaths and the person-years (PY) at deaths. There was a difference in the proportion of deaths and the person-years according to income group (1st quintile: the proportion = 67.2%, PY = 0.670; 2nd quintile: the proportion = 62.2%, PY = 0.669; 3rd quintile: the proportion = 66.9%, PY = 0.852; 4th quintile: the proportion = 62.8%, PY = 0.725; 5th quintile: the proportion = 60.9%, PY = 0.727).

**Table 1:** Patient characteristics, number of deaths, and mean survival periods

Variables	N/Mean	%/SD	Number of deaths	%	p*	Incidence per person-years
Sex						
Male	4658	63.6	1491	32%	<.001	0.768
Female	2667	36.4	576	22%		0.819
Age group						
49 or younger	2903	39.6	391	13%	<.001	0.726
50–59	1772	24.2	395	22%		0.704
60–69	1574	21.5	600	38%		0.769
70 or older	1076	14.7	681	63%		0.888
Income (quintiles)						
The 5th (highest)	2197	30.0	549	25%	<.001	0.810
The 4th	1649	22.5	429	26%		0.720
The 3rd	1310	17.9	371	28%		0.774
The 2nd	1083	14.8	339	31%		0.821
The 1st (lowest)	1086	14.8	379	35%		0.789
Disability						
None	6751	92.2	1824	27%	<.001	0.791
Mild	459	6.3	189	41%		0.725
Severe	115	1.6	54	47%		0.673
New cases by year						
2004	1478	20.2	450	30%	0.033	0.822
2005	1493	20.4	433	29%		0.776
2006	1324	18.1	387	29%		0.773
2007	1352	18.5	362	27%		0.819
2008	1678	22.9	435	26%		0.727
Hepatitis B						
No	4749	64.8	1397	29%	0.002	0.822
Yes	2576	35.2	670	26%		0.708
Hepatitis C						
No	5897	82.2	1730	29%	0.036	0.815
Yes	1304	17.8	337	26%		0.643
Alcoholic liver cirrhosis						
No	6820	93.1	1786	26%	<.001	0.810
Yes	505	6.9	281	56%		0.637
Other (non-viral, non-alcoholic) liver cirrhosis						
No	6895	94.1	1901	28%	<.001	0.801
Yes	430	5.9	166	39%		0.610
Regional Level						
Current smoking rate^†^	23.6	3.0				
High-risk drinking rate^†^	16.4	3.7				
Walking exercise practice rate ^†^	51.8	12.2				
Obesity rate ^†^	21.5	2.9				
Percentage of college graduates ^†^	34.2	12.6				

*Chisq-test ^†^Mean/SD

**Table 2:** The proportion and survival time according to income of the death

Variables	Income (quintiles)										
	The 1st (lowest)		The 2nd		The 3rd		The 4th		The 5th (highest)		p*
	%	PY †	%	PY ^†^	%	PY ^†^	%	PY ^†^	%	PY ^†^	
Sex											
Male	39.4	0.763	37.0	0.853	33.4	0.776	29.8	0.703	26.8	0.772	0.094
Female	27.8	0.854	20.8	0.729	19.4	0.769	18.8	0.779	22.0	0.898	
Age group											
49 or younger	18.2	0.963	17.6	0.975	14.6	0.851	12.0	0.731	9.6	0.935	<.001
9	29.1	0.781	28.3	0.908	22.4	0.674	22.7	0.770	15.5	0.774	
60–69	41.8	0.701	41.1	0.760	45.2	0.801	36.1	0.639	31.6	0.662	
70 or older	67.2	0.670	62.2	0.669	66.9	0.852	62.8	0.725	60.9	0.727	
Disability											
None	33.6	0.820	30.2	0.823	27.0	0.767	25.0	0.736	23.9	0.819	<.001
Mild	44.8	0.747	40.8	0.742	41.4	0.761	38.1	0.611	41.0	0.792	
Severe	44.8	0.453	52.6	1.039	59.1	1.088	36.4	0.647	43.5	0.568	
New cases by year											
2004	37.2	0.816	28.1	0.765	34.9	0.697	28.9	0.907	27.2	0.899	0.002
2005	39.2	0.687	34.0	0.947	29.7	0.828	25.7	0.745	23.9	0.729	
2006	35.6	0.925	35.5	0.883	28.1	0.732	28.7	0.618	24.8	0.839	
2007	30.7	0.911	33.0	0.734	27.6	0.944	22.1	0.635	24.5	0.921	
2008	33.0	0.729	26.3	0.774	22.8	0.725	24.4	0.720	24.4	0.708	
Hepatitis B											
No	36.1	0.851	30.9	0.826	28.8	0.820	27.9	0.729	26.9	0.885	0.426
Yes	32.7	0.686	32.1	0.810	27.4	0.700	22.8	0.702	21.4	0.672	
Hepatitis C											
No	35.4	0.818	31.0	0.916	28.4	0.813	27.0	0.729	25.7	0.837	0.355
Yes	32.4	0.658	32.8	0.553	27.9	0.639	21.7	0.680	21.6	0.686	
Alcoholic liver cirrhosis											
No	32.3	0.854	28.3	0.860	25.6	0.782	24.0	0.737	24.2	0.834	<.001
Yes	62.1	0.560	63.7	0.671	56.6	0.740	51.6	0.638	44.0	0.579	
Other (non-viral, non-alcoholic) liver cirrhosis											
No	33.6	0.809	30.6	0.831	27.7	0.803	25.6	0.751	24.5	0.818	0.420
Yes	53.6	0.643	42.9	0.715	37.5	0.551	32.1	0.491	33.9	0.715	

*Chisq-test, ^†^ Incidence per person-years

### Risk factors associated with mortality in HCC

Table 3 shows the hazard ratios of patients with HCC according to both Cox proportional hazard models after adjusting for all other covariates. The HRs of HCC patients increased with age and lower income (*p* < 0.001). However, there were no significant differences between those in the 4th and 5th quintiles of income (*p* = 0.161). Furthermore, HCC patients with HBV, alcoholic liver cirrhosis, and other types of liver cirrhosis had higher HRs than those without these conditions (HBV: HRs =1.172, *p* = 0.001; alcoholic liver cirrhosis: HRs = 2.187, *p* < 0.001; other liver cirrhosis: HRs = 1.214 *p* = 0.023), but the opposite pattern was found with regard to HCV (HRs = 0.812, *p* < 0.001). The consequences of community factors indicated that higher current smoking rates and a greater percentage of college graduates in the community were associated with higher HRs, and that walking for exercise was associated with lower HRs among HCC patients.

**Table 3:** Adjusted hazard ratios of hepatocellular carcinoma mortality by multi-level

Variables	HR(Hazard Ratio)	*p*-value
Sex		
Female	Reference	
Male	1.694	<.001
Age group		
49 or younger	Reference	
50–59	1.691	<.001
60–69	3.338	<.001
70 or older	8.267	<.001
Income (quintiles)		
The 5th (highest)	Reference	
The 4th	1.096	0.161
The 3rd	1.323	<.001
The 2nd	1.414	<.001
The 1st (lowest)	1.451	<.001
Disability		
None	Reference	
Mild	1.144	0.084
Severe	1.454	0.008
New cases by year		
2004	Reference	
2005	0.949	0.441
2006	1.030	0.675
2007	0.933	0.336
2008	0.914	0.192
Hepatitis B		
No	Reference	
Yes	1.172	0.001
Hepatitis C		
No	Reference	
Yes	0.812	<.001
Alcoholic liver cirrhosis		
No	Reference	
Yes	2.187	<.001
Other (non-viral, non-alcoholic) liver cirrhosis		
No	Reference	
Yes	1.214	0.023
Regional Level		
Current smoking rate*	1.019	0.038
High-risk drinking rate*	0.994	0.331
Walking exercise practice rate *	0.996	0.037
Obesity rate *	1.003	0.634
Percentage of college graduates *	1.005	0.023
Income^†^	1.106	<.001

*Continuous variable at regional level ^†^ Trend test according to decrease in income level

### Subgroup analyses: HCC mortality by sex and age

Table 4 presents the results of multilevel multivariate analyses of HCC mortality by sex and age. Among male HCC patients, there was a difference in hazard ratio according to income group (1st quintile: HR =1.422, *p* < 0.001; 2nd quintile: HR =1.560, *p* < 0.001; 3rd quintile: HR =1.422, *p* < 0.001; reference group, 5th quintile). Among HCC patients aged 50–59, there was a difference in hazard ratio according to income group (4th quintile: HR =1.509, *p* = 0.010; 3rd quintile: HR =1.593, *p* = 0.005; 2nd quintile: HR =2.089, *p* < 0.001; 1st quintile: HR =2.197, *p* < 0.001; reference group, 5th quintile), and a significant association also was found in those aged 49 years or younger and 60–69. However, there was no such association among women or those aged 70 years or older who had been diagnosed with HCC.

**Table 4:** Adjusted hazard ratios of hepatocellular carcinoma mortality by sex and age groups

Variables		Hazard ratio	95% Hazzard ratio confidence limits		*p*-value
Male	Income (quintiles)				
	The 5th (highest)	Reference			
	The 4th	1.140	0.979	1.328	0.092
	The 3rd	1.422	1.212	1.669	<.001
	The 2nd	1.560	1.325	1.838	<.001
	The 1st (lowest)	1.541	1.309	1.815	<.001
	Trend test^†^	1.127	1.087	1.169	<.001
Female	Income (quintiles)				
	The 5th (highest)	Reference			
	The 4th	1.033	0.810	1.318	0.794
	The 3rd	1.107	0.857	1.429	0.436
	The 2nd	1.129	0.862	1.477	0.379
	The 1st (lowest)	1.265	0.995	1.607	0.055
	Trend test^†^	1.057	1.000	1.118	0.049
Age 49 or younger	Income (quintiles)				
	The 5th (highest)	Reference			
	The 4th	1.270	0.929	1.735	0.135
	The 3rd	1.595	1.166	2.181	0.004
	The 2nd	1.714	1.239	2.371	0.001
	The 1st (lowest)	1.568	1.121	2.193	0.009
	Trend test^†^	1.131	1.052	1.215	0.001
Age 50–59	Income (quintiles)				
	The 5th (highest)	Reference			
	The 4th	1.509	1.105	2.059	0.010
	The 3rd	1.593	1.151	2.204	0.005
	The 2nd	2.089	1.512	2.887	<.001
	The 1st (lowest)	2.197	1.586	3.043	<.001
	Trend test^†^	1.209	1.126	1.298	<.001
Age 60–69	Income (quintiles)				
	The 5th (highest)	Reference			
	The 4th	1.116	0.875	1.423	0.377
	The 3rd	1.474	1.147	1.895	0.003
	The 2nd	1.355	1.036	1.772	0.026
	The 1st (lowest)	1.354	1.044	1.757	0.022
	Trend test^†^	1.087	1.026	1.150	0.004
Age 70 or older	Income (quintiles)				
	The 5th (highest)	Reference			
	The 4th	0.967	0.777	1.202	0.759
	The 3rd	1.140	0.894	1.454	0.292
	The 2nd	1.074	0.841	1.372	0.569
	The 1st (lowest)	1.177	0.945	1.466	0.146
	Trend test^†^	1.042	0.991	1.096	0.111

*All adjusted by sex, age group, disability, new cases by year, hepatitis B, hepatitis C, alcoholic liver cirrhosis, other (non-viral, non-alcoholic) liver cirrhosis and regional level(current smoking rate, high-risk drinking rate, walking exercise practice rate, obesity rate, percentage of college graduates). ^†^Trend test according to decrease in income level

Table 5 presents the adjusted HRs of HCC mortality for the lowest (1st quintile) and highest (5th quintile, reference) income groups by sex and age considering group interactions. Among male patients, the lowest income group was associated with an increased risk for HCC mortality compared to the highest income group among patients 50–59 years and 49 years or younger (HR = 1.956, *p* < .001 for 49 or younger; HR = 2.678, *p* < .001 50–59 years). No such association was observed among middle aged female patients (*p* = 0.151 for 49 or younger; *p* = 0.734 50–59 years).

**Table 5:** Adjusted hazard ratios of hepatocellular carcinoma mortality between low- and high-income groups by sex and age groups by testing interactions

Variable			Hazzard ratio	95% Hazzard ratio confidence limits		*P*-value
Male	Age 49 or younger	Income (quintiles)				
		The 5th (highest)	Reference			
		The 1st (lowest)	1.956	1.341	2.854	<.001
	Age 50–59	Income (quintiles)				
		The 5th (highest)	Reference			
		The 1st (lowest)	2.678	1.827	3.924	<.001
	Age 60–69	Income (quintiles)				
		The 5th (highest)	Reference			
		The 1st (lowest)	1.240	0.913	1.683	0.169
	Age 70 or older	Income (quintiles)				
		The 5th (highest)	Reference			
		The 1st (lowest)	1.132	0.836	1.534	0.423
Female	Age 49 or younger	Income (quintiles)				
		The 5th (highest)	Reference			
		The 1st (lowest)	0.529	0.221	1.262	0.151
	Age 50–59	Income (quintiles)				
		The 5th (highest)	Reference			
		The 1st (lowest)	1.120	0.582	2.157	0.734
	Age 60–69	Income (quintiles)				
		The 5th (highest)	Reference			
		The 1st (lowest)	1.678	1.007	2.796	0.047
	Age 70 or older	Income (quintiles)				
		The 5th (highest)	Reference			
		The 1st (lowest)	1.218	0.875	1.697	0.243

*All adjusted by sex, age group, disability, new cases by year, hepatitis B, hepatitis C, alcoholic liver cirrhosis, other (non-viral, non-alcoholic) liver cirrhosis and regional level(current smoking rate, high-risk drinking rate, walking exercise practice rate, obesity rate, percentage of college graduates)

## Discussion

Having a low income significantly affected the overall 5-year mortality of Korean adults newly diagnosed with HCC from 2004 to 2008. Middle-aged men with HCC were more vulnerable to the effects of low income on 5-year mortality than were younger and older men and compared to women of all ages.

Our results are similar to those of previous research on the association between health outcomes and SES among HCC patients [20–22]. Although it is difficult to compare health outcomes across health systems, HCC patients living in economically deprived areas in the U.S. are more likely to be diagnosed at an earlier age [20], and those living in the U.K. have a shorter life expectancy [21]. According to nationally representative U.S. cancer registry data, health insurance type (uninsured and Medicaid) and living in low-income communities are associated with worse health outcomes in HCC patients [23]. The importance of monitoring and screening populations at risk for HCC, particularly young adults with HBV and/or intravenous drug users, cannot be stressed enough. Economic deprivation and poor access to healthcare likely result in a greater risk for HCC and a shorter survival time. Moreover, among U.S. adults with chronic liver disease (CLD), low income contributed to an increased risk for liver-related mortality [24].

Sudden loss of wealth or a home has been shown to constitute major psychological stressors among U.S. adults [25–27]. Low-income adults with HCC might not be able to afford surcharged services, such as specialty doctors at general hospitals and new and high-cost technology; they also might show lower adherence to prescribed medication regimens and delay needed medical care during the early stages of HCC beyond the NHI coverage deadline [8, 27]. In other words, due to the possible burden of high out-of-pocket expenses, low-income HCC patients might not be able to benefit from new and high-cost diagnostic and therapeutic technology that is not covered by the NHI [11, 12]. The effects of low income on HCC mortality can extend to non-medical domains, particularly among middle-aged adults. Indeed, during the Great Recession of the late 2000s in the U.S., non-medical social welfare spending provided a social safety net for middle-aged individuals, who generally make larger economic contributions but receive fewer welfare benefits compared to older individuals [28]. The effects of low income on HCC mortality often decrease in later life because of the increased availability of social welfare programs and access to health care with lower amounts of out-of-pocket expenses observed in older individuals [1, 3, 29]. Health behaviors are plausible mediators of health disparities because of social patterning and these influences on health outcomes [30]. Among socially disadvantaged individuals, for example, low-income individuals are prone to be more influenced by sudden loss of wealth or a home, perception of fewer benefits of health behaviors, and pessimistic attitudes of later life [30].

This study had several limitations, and caution is required when interpreting the results and attempting to generalize its findings. Although we analyzed all nationwide inpatient claims for HCC during a defined period, Korea’s unique healthcare delivery and insurance system might significantly limit generalizability of the results to other nations. In addition, given the nature of the health insurance claims dataset, this study retrospectively calculated the time of diagnosis of HCC patients. Although we used the diagnostic information in the claims data, we are confident that the time of diagnosis used in this study reflects the time of actual diagnosis of HCC patients because we reviewed the claims in all available years and excluded the first 2 years of data. However, some degree of measurement error due to unavailability of data on the actual time of diagnosis was unavoidable. Therefore, additional research using cohort data should be performed to verify the associations examined in this study. In addition, potentially important clinical information was not available. For example, we were not able to access the detailed clinical information on HCC patients contained in the health insurance claims data collected by the National Cancer Center. Although we included duration from diagnosis to death or end of follow up in the analytic models, additional clinical information would have improved the validity of our findings. Additional information, such as cancer stage, site of cancer, and type of cancer, should be considered in future studies to build on our findings and calibrate estimates of the survival probability of HCC patients. In addition, detailed individual- and community-level information on SES was not available for our analyses. For example, it might have been helpful to include educational attainment and income inequality indicators by geographic unit because these can affect the health of both poor and wealthy individuals due to spillover effects (e.g., psychological stress) of income inequality, which can result in erosion of social cohesion [31, 32]. Additional studies should be conducted using a dataset with more detailed matching of NHI claims data as well as more information on SES.

Despite these limitations, to the best of our knowledge, this is the one of only a few studies to analyze the Korean national claims dataset of HCC patients and to explore individual- and community-level factors associated with the survival probability of these individuals.

## Conclusions

Having a low income significantly affected the overall 5-year mortality of Korean adults who were newly diagnosed with HCC from 2004 to 2008. Middle-aged men were the most vulnerable. We believe our findings will be useful to healthcare policymakers in Korea as well as to healthcare leaders in countries with NHI programs who need to make important decisions about allocation of limited healthcare resources according to a consensually accepted and rational framework. Our findings also add to the mounting empirical support for development of a national cancer management strategy to narrow the gaps in, for example, survival time and access to healthcare according to demographic characteristics, including SES.

## Data Availability

The data generated by the National Health Insurance Corporation, Republic of Korea, are not publicly available.

## Abbreviations

HCC: Hepatocellular Carcinoma
HR: Hazard Ratio
HBV: Hepatitis B
HCV: Hepatitis C
NHI: National Health Insurance
SES: Socioeconomic Status
NHIS-NSC: Korean National Health Insurance Service-National Sampling Cohort
KCHS: Korea Community Health Survey
ICD-10: International Classification of Diseases, Version 10
BMI: Body Mass Index
PY: Person-Years
CI: Confidence Interval
CLD: Chronic Liver Disease
NRF: National Research Foundation of Korea
